# Species-Specific Responses of Root Morphology of Three Co-existing Tree Species to Nutrient Patches Reflect Their Root Foraging Strategies

**DOI:** 10.3389/fpls.2020.618222

**Published:** 2021-01-25

**Authors:** Zhenya Yang, Benzhi Zhou, Xiaogai Ge, Yonghui Cao, Ivano Brunner, Jiuxi Shi, Mai-He Li

**Affiliations:** ^1^Research Institute of Subtropical Forestry, Chinese Academy of Forestry, Hangzhou, China; ^2^Zhejiang Provincial Key Laboratory of Bamboo Research, Zhejiang Academy of Forestry, Hangzhou, China; ^3^Qianjiangyuan Forest Ecosystem Research Station, National Forestry and Grassland Administration, Hangzhou, China; ^4^Forest Soils and Biogeochemistry, Swiss Federal Research Institute WSL, Birmensdorf, Switzerland; ^5^Forest Dynamics, Swiss Federal Research Institute WSL, Birmensdorf, Switzerland; ^6^Key Laboratory of Geographical Processes and Ecological Security in Changbai Mountains, Ministry of Education, School of Geographical Sciences, Northeast Normal University, Changchun, China

**Keywords:** nutrient patch, foraging strategy, root plasticity, root response ratio, non-structural carbohydrates

## Abstract

Root foraging strategies of plants may be critical to the competition for nutrient resources in the nutrient patches, but little is known about these of co-existing tree species in subtropical regions. This study aimed to elucidate root foraging strategies of three co-existing tree species in nutrient heterogeneous soils by exploring their root distribution, root morphology, photosynthates allocation and nutrient accumulation. Seedlings of the three tree species [moso bamboo (*Phyllostachys edulis*), Chinese fir (*Cunninghamia lanceolata*), and masson pine (*Pinus massoniana*)] were grown for 8months under one homogeneous soil [uniform nitrogen (N) plus phosphorus (P)] and three heterogeneous soils (localized N supply, localized P supply, or localized N plus P supply). The biomass, root morphological parameters (i.e., root length and root surface area), specific root length (SRL), non-structural carbohydrates (NSCs, i.e., mobile sugar and starch) in roots, total N and total P of plants were measured. The plasticity and distribution of root system were analyzed by calculating the root response ratio (RRR) and root foraging precision (FP), respectively. The results are as follows (i) Chinese fir tended to forage more N by promoting root proliferation in the N-rich patch, while root proliferation of bamboo and pine did not change. For P, bamboo absorbed more P by promoting root proliferation in the P-rich patch. The total P content of Pine and Chinese fir under localized P supply treatment remain the same despite the fact that the root length in the P-rich patch and the FP increased. (ii) Chinese fir foraged more N by increasing root length and decreasing SRL in the NP-rich patch; bamboo foraged more N and P by increasing root length and SRL in the NP-rich patch. The FP and foraging scale (FS) of both bamboo and Chinese fir were significantly improved under localized N plus P treatment. (iii) The concentrations of NSC were positively correlated with root morphological plasticity for moso bamboo and Chinese fir. Our results indicated that higher morphological plasticity is exhibited in moso bamboo and Chinese fir than masson pine in nutrient heterogeneous soils, allowing them to successfully forage for more nutrients.

## Introduction

The distribution of nutrients in soil is typically highly heterogeneous over space and time (Caldwell et al., 1991; Cheng et al., 2016). Heterogeneous nutrient condition has been proposed to influence root growth (Hutchings and Kroon, 1994; Lynch et al., 2001; Cheng et al., 2016), nutrient uptake (Li et al., 2014, 2017), and competitive outcomes among species (Rajaniemi and Reynolds, 2004; Chen et al., 2018). The morphological and physiological plasticity will evolve enabling plants to cope with, and perhaps even to benefit from heterogeneous nutrient conditions (Wijesinghe and Hutchings, 1999; Lark et al., 2004; Li et al., 2017).

The behavior of plants involved in exploring, obtaining and utilizing resources through a series of morphological and physiological changes of the root are defined as the root foraging strategy (Einsmann et al., 1999; de Kroon et al., 2005). Several studies confirmed that plants are capable of proliferating more roots in nutrient-rich patches to uptake nutrients more effectively (Robinson, 1994; Fransen and de Kroon, 2001). Some tree species maximize the proportion of roots in nutrient-rich patches by reducing the distribution of roots in nutrient-deficient patches, thereby improving their roots foraging precision (FP; the ability to concentrate roots in rich-nutrient patches; Drew, 1975; Granato and Raper, 1989; Campbell et al., 1991; Hodge, 2009; Chen et al., 2016). The size and mode of root morphological plasticity of plants in heterogeneous soil strongly depend upon the plants themselves (Robinson, 1994; Pablo et al., 2012). For instance, growth rate (Van de Vijver et al., 1993; Fransen et al., 1999), root thickness (Hodge, 2004; Liao et al., 2014), sensitivity to nutrient elements (Zhang et al., 2013; Li et al., 2014, 2017), and mycorrhizal types (Hodge and Fitter, 2010; Comas et al., 2014; Valverde-Barrantes et al., 2017) have all been reported to affect the root plasticity of plant in heterogeneous soil. Such a difference among tree species is of great ecological value in the coexistence of plants and the maximization of soil use efficiency (Fransen and de Kroon, 2001; James et al., 2009; Shen et al., 2013). Competition between/among species will intensify when co-existing plants adopt the same foraging strategy (Eissenstat and Caldwell, 1988; Adams et al., 2013). Root foraging strategies will be more complex when various elements are involved in nutrient patches (Shen et al., 2011; Jing et al., 2012; Li et al., 2014; Huysen et al., 2016). Therefore, exploring the foraging strategies of co-existing tree species in heterogeneous nutrient conditions is beneficial to alleviate the competition among tree species and improve the utilization efficiency of soil space.

Based solely on the amount of roots, the foraging strategies of roots in the nutrient patch do not seem to be sufficiently elucidated. The carbon investment of root and root formation strategy should also be considered, such as the change in specific root length (SRL) and non-structural carbohydrates (NSCs) concentration. The increase in the NSC concentration at a growth site can improve the flexibility of the plant growth in response to fluctuating environments (Li et al., 2013; Villar-Salvador et al., 2015; Chen et al., 2017b). It has been extensively demonstrated that SRL and NSCs exhibit species-specific flexible plasticity in dynamic nutrient environments (Lei et al., 2013; Zhang et al., 2013; Li et al., 2017; Yan et al., 2019). However, SRL and NSCs in responses to nutrient heterogeneity soil have not been studied as root formation strategies and foraging strategies. The availability of rhizosphere nutrients leads to a change in the whole root morphology (Giehl et al., 2014). Speed and efficiency of nutrient uptake of co-existing plants determine their competitiveness and productivity (Orman-Ligeza et al., 2013; Giehl and Wirén, 2014). In addition to root distribution, root architecture, root length density, and mycorrhizal traits, the rate of root nutrient uptake also reflects the foraging ability of roots to some extent (Chen et al., 2016; Wang et al., 2016). However, conflicting results have been reported about the effect of root morphological plasticity in response to nutrient heterogeneous soils on accumulation of nutrients and total biomass (Nakamura et al., 2008; He et al., 2012; Li et al., 2014; Wang et al., 2018). Exploring the contribution of root morphological plasticity to nutrient accumulation may explain the difference in the nutrient competitiveness of co-existing trees. The foraging ability of roots will inevitably affect the growth of the aboveground plant parts, and in turn, the supply of photosynthates (e.g., NSCs) from the aboveground parts will certainly affect the development of roots (Jackson et al., 1990; Fransen et al., 1999; Eissenstat et al., 2000; Liu et al., 2018). Therefore, the carbon input of the root system, root morphological plasticity, and nutrient uptake should be studied as a system, which is often ignored or separated in previous studies.

Moso bamboo (*Phyllostachys edulis*), Chinese fir (*Cunninghamia lanceolata*), and masson pine (*Pinus massoniana*) co-exist often as mixed forest in subtropical China, and they present different root systems (taproot or fibrous root) and different growth rates. Therefore, the three tree species may exhibit different foraging strategies in the process of adapting to heterogeneous soil, and this difference could be used to improve the coexistence and soil utilization efficiency (Caldwell et al., 1991; Shen et al., 2013). Both Chinese fir and masson pine roots were found to grow better in heterogeneous phosphorus (P) soils than in P deficient soils and to exhibit species-specific foraging tendencies in response to nitrogen (N) supply heterogeneity of horizontal distribution. And the foraging efficiency of masson pine with different genotypes varied greatly (Wu et al., 2011; Zhang et al., 2013; Yan et al., 2020). However, the foraging strategies of masson pine and Chinese fir in nutrient patches have not been revealed systemically, such as root distribution, root formation, and nutrient absorption. Moso bamboo distributes more roots in the upper soil layer in the process of nutrient foraging to squeeze the roots of broad-leaved trees into the deep soil space (Shen et al., 2016). We aimed to study the foraging strategies of three tree species in nutrient patches, through investigating the responses of root distribution, root morphology, nutrient uptake and photosynthates allocation to nutrient heterogeneous soil. The hypotheses tested in this study were that (1) the root foraging strategies of the three tree species are species-specific and nutrient-specific, (2) the root plasticity of fibrous root species is higher than that of taproot species, and (3) root morphological plasticity is closely related to the concentration of NSC in roots.

## Materials and Methods

### Experimental Design

The experiment was carried out in a greenhouse with controlled temperature (25°C daytime, 20°C night time) and relative air humidity (75%) and ambient light. The experimental soil with a low nutrient content was taken from Qianjiangyuan Forest Ecosystem Research Station (Hangzhou, 119°95'E, 29°48'N). The soil (barren acidic red soil from the Fuyang District of Zhejiang Province) passed through a mesh sieve (aperture 2mm) after being air-dried. One kilogram of this soil (pH = 4.91) contained 18.7g of organic C, 0.86g of total N, 0.26g of total P, 11.2g of total K, 85.1mg of hydrolysable N, 4.15mg of available P, and 65.7mg of available K.

Three typical subtropical Chinese tree species, moso bamboo, Chinese fir, and masson pine, often co-existing in the nature, were selected for this experiment. The experimental seeds of each species were selected from the same parent plant. The parent plants of bamboo, Chinese fir and pine are located in Da Jing town forestry center (Guilin, Guangxi Province), Changle forestry center (Hangzhou, Zhejiang Province) and Laoshan forestry center (Hangzhou, Zhejiang Province) respectively. In March, typical seeds of each species (from the same provenance) were germinated on wet filter paper with deionized water at 25°C in an incubator until budding. Seedlings of similar radicle length were planted in a seedling disk. For each species, 42 seedlings with similar heights (bamboo 7.8cm, Chinese fir 6.2cm, and pine 5.3cm) and root lengths (bamboo 5.4cm, Chinese fir 5.0cm, and pine 4.8cm) were selected for planting in experimental containers after the leaves emerged.

**Figure 1:**
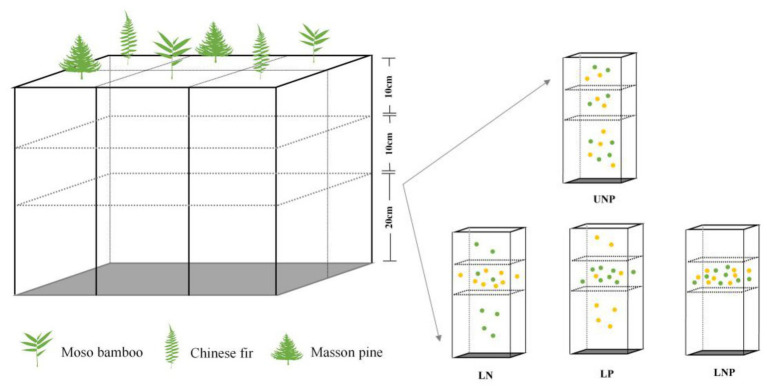
Schematic drawing of the container and the experimental treatments. Each treatment was repeated six times (i.e., container), and two seedlings of each species were randomly planted into each container with two replicates. Each yellow point denotes 0.25g N, and each green point denotes 0.25g P. UNP, uniform N plus P supply; LN, localized N supply; LP, localized P supply; and LNP, localized N plus P supply. The same below.

Plastic boxes with suitable air permeability and strength were used as containers for the experiment (Figure 1). The walls of the plastic boxes were evenly perforated with small holes of the same size for air permeability and water addition, and a uniform shading yarn was attached to the inner wall of the containers to reduce moisture evaporation. The plastic boxes (61cm × 41cm × 41cm) were divided into six cuboids of equal volume (20cm × 20cm × 41cm) separated by PVC plates, and each cuboid was divided into three layers: the first layer was 0–10cm, the second layer was 10–20cm, and the third layer was 20–40cm (Figure 1).

Four different nutrient supply treatments were applied: (1) UNP: uniform N plus P supply throughout the three soil layers; (2) LN: localized N supply in the 10–20cm layer; (3) LP: localized P supply in the 10–20cm layer; and (4) LNP: localized N plus P supply in the 10–20cm layer (Figure 1). We compared localized fertilization treatment (LN, LP, and LNP) with UNP to understand the root responses to localized nutrient supply when the same amount of nutrients was added (Figure 1). One of the purposes of this study was to judge the changes in indicators related to root morphological plasticity and soil nutrients distribution, such as total biomass, nutrient accumulation, and NSCs concentration. Hence, UNP was chosen as the control to ensure equal total nutrients amount in the four treatments to avoid the interference of total nutrients change to our judgment. The 10–20cm layer of LN, LP, and LNP treatments was considered as N-rich patch, P-rich patch, and NP-rich patch, respectively. The distribution of N and P in soil is often vertically stratified due to the subsidence of N with water fall during drought and the poor mobility of P (Lynch, 2013). Therefore, soil nutrient heterogeneity was designed in vertical direction. Compared with putting roots in nutrient hotspot at the beginning of roots development, an appropriate distance between nutrient-rich areas and primary root is preferred to estimate the roots ability of exploring nutrients patch. Therefore, nutrient-rich patches were designed in 10–20cm soil layer referring to the classical experimental method of Drew (1975).

Urea (CH_4_N_2_O) as the N source and calcium perphosphate monohydrate [Ca(H_2_PO_4_)_2_ · CaSO_4_ · H_2_O] as the P source were used for nutrient treatments. The available P content (P_2_O_5_) of calcium perphosphate monohydrate is approximately 14.5%. In the UNP treatment, 2g N and 2g P were uniformly distributed throughout the 0–40cm soil column. In the LN treatment, 2g N was applied to the 10–20cm layer, while 2g P was evenly distributed throughout the 0–40cm soil column. In the treatment LP, 2g P was applied to the 10–20cm layer, while 2g N was distributed throughout the 0–40cm soil column evenly. In the treatment LNP, 2g of N and P each were applied to the 10–20cm layer (nutrient-enriched layer; Figure 1). Fertilizer added at the above dose was found to bring available P and hydrolyzed N in soil to the rich level (level 2, according to national grading standards for soil nutrient content) and to promote the growth of all the three species significantly in pre-experiment on uniform fertilization. Untreated soil was classified nutrient-deficient (the hydrolyzed N reaches level 4, while available P reaches just level 5), while nutrient content in nutrient-enriched layer reaches extremely rich level (level 1; Wang, 2015). Fertilizer and soil were mixed according to the nutrient treatments mentioned above and before the soil layers were filled into the cuboids. Soils or soil-nutrient mixtures were carefully added to each layer (40–20, 20–10, and 10–0cm), and approximately 20kg soil or soil-nutrient mixtures were filled into each cuboid (Figure 1). In total, 12 plastic containers were used, with three containers randomly selected for each treatment. Six seedlings (two seedlings per species and one seedling per cuboid) were randomly planted in the top layer of each plastic container in April (Figure 1). Each individual was regarded as a replicate; hence, there was a total of six replicates (three boxes per treatment × two seedlings in each box = 6). Each container was rotated clockwise by 90° monthly to minimize the micro-environmental effects on individuals inside the greenhouse (Pinto et al., 2011).

To prevent nutrient movement from top-down leaching when watering, distilled water was injected into each layer (0–10, 10–20, and 20–40cm) with a 200ml syringe through lateral holes on the upper third of each layer weekly. The amount of water added to each 10-cm thick soil layer was equal among all treatments, and the maximum amount of water added was ≤80% of the maximum field water holding capacity of the soil. Soil moisture of each layer was measured by a soil moisture measurement system (Aozuo ecological instrument, Trime-pico AZS-100, Germany) through the small holes in the container wall before adding water.

### Harvest and Measurements

After 8-month’s growth, all seedlings were harvested in November. The aboveground parts were separated at the soil surface and put into numbered self-sealing bags, and then the roots were very carefully separated layer by layer according to the treatments shown in Figure 1. The small and scattered roots of each soil layer were collected with a mesh sieve (aperture 4mm). All samples were preserved in an ice-box and brought back to the laboratory for storage in a 0–2°C refrigerator for no more than 1h before scanning.

The roots were cleaned with distilled water and dried with absorbent paper. Then, the roots were scanned with a double-sided scanner at a resolution of 500 dpi (Regent Instruments Inc., WinRhizo Pro, Canada). Root images were analyzed using WinRhizo software to obtain root parameters, such as root length and root surface area. After scanning, the roots, stems, and leaves were devitalized at 105°C for 30min (to minimize the physiological activity; Li et al., 2018) and then dried at 65°C to a constant weight to obtain the dry weight (biomass) of the tissues. After grinding samples with high-throughput tissue grinder (Retsch GmbH, MM400, Germany), the concentrations of N and P in roots, stems, and leaves were measured by the H_2_O_2_-H_2_SO_4_ method and Vanadium molybdenum yellow colorimetry, and the concentration of mobile sugars and starch was measured by anthrone colorimetry (Li et al., 2008). At the end of the experiment, we detected the soil nutrients content and found that the available P and hydrolyzed N content in the 10–20cm layer soil remained the rich level.

### Calculations and Statistics

The total N or P accumulation per plant is the sum of N or P in leaves, stems, and roots of a plant, and the N or P in each tissue was calculated using the tissue biomass multiplied by the N or P concentration in that tissue. The average nutrients content were calculated by dividing the total nutrient content by the total biomass. The concentration of NSCs (%) is defined as the sum of the concentration of sugars plus starch in that tissue (Li et al., 2018). The SRL (mg^−1^) is expressed as the length per unit dry weight of root, reflecting the cost inputs of the root growth and formation strategy (Eissenstat and Caldwell, 1988; Poorter and Ryser, 2015).

The plasticity of different tree species to localized nutrients can be quantitatively estimated using the root response ratio (RRR), which was defined as the ratio of the extent of root change in the nutrient-enriched zone (10–20cm layer) to that in the same 10–20cm layer in the uniform nutrient supply treatment (UNP treatment; Figure 1; Li et al., 2014):

RRR=∑xi'j'−xij/xi'j'+xij/n

where xi'j' is the root length in the nutrient-enriched zone (10–20cm layer) and xij is the root length in the 10–20cm layer under UNP treatment (Figure 1). Both j and j' are replicates (j = 1, 2,… 6) of each species. The i' indicates the localized nutrient treatments, and i is related to UNP. n is the number of xi'j'-xij values. In the present study, *n* is equal to 36 because six replicates (six random individuals) were set in each pair of nutrient treatments. Moreover, we calculated and analyzed the response ratio of the total root length (RRRt) across the 0–40cm soil layer instead of the RRR for the 10–20cm soil layer. The RRRt is, therefore, the total RRR for the localized nutrient enrichment:

RRRt=∑xti'j'−xtij/xti'j'+xtij/n

where xti'j' is the total root length (0–40cm soil layer) under localized fertilization treatments (LN, LP, or LNP), xtij is the total root length (0–40cm soil layer) under the uniform nutrient supply treatment (UNP), and n is 36. The calculation method refers to method of Valladares et al. (2006; relative distance plasticity index) for quantitative analysis of phenotypic plasticity. This method is sensitive to balancing the plasticity norms of different genotypic tree species and can be used to compare phenotypic plasticity among different species.

We also calculated the foraging precision of three species under four treatments:

$$\mathrm{FP}=\left({\mathrm{RL}}_{\mathrm{rich}}-{\mathrm{RL}}_{\mathrm{poor}}\right)/{\mathrm{RL}}_{\mathrm{total}}$$

where FP is the foraging precision, RL_rich_ is usually defined as the root length in nutrient enrichment layer, RL_poor_ is the root length in nutrient-poor layer, and RL_total_ is the total root length (Chen et al., 2018). Obviously, for the three localized fertilization treatments, the RL_rich_ of each sampling refers to the root length in the 10–20cm layer of the cuboid, while the RL_poor_ the sum of the root length in the 0–10 and 20–40cm layers. The RL_rich_ of UNP (the control group) should also be the root length in the 10–20cm layer and the RL_poor_ of UNP is the sum of the root length in the 0–10 and 20–40cm layers. Foraging scales (FS) of three species under different treatments can be represented by total root length (Grime, 2007).

Three-way ANOVA was performed to test the effects of species, nutrient treatments, soil layers, and their interactions on root parameters (the data come from different soil layers; Table 1). Thus, we subsequently used two-way ANOVA, one-way ANOVA, and *post hoc* tests to analyze the differences among species and treatments (considering the whole root system or the 10–20cm soil layer). The correlation between root morphological and nutrient accumulation as well as the NSC concentration was analyzed by Pearson’s correlation analysis. All data were tested for normality (Kolmogorov-Smirnov and Shapiro-Wilk test, *p* > 0.05) before correlation analysis. SPSS statistical software was used for all analyses. Origin 9.0 and Excel 10 were used to construct all figures and tables.

**Table 1:** Effects of species, nutrient treatments, soil layers, and their interactions on root parameters.

Parameter	Species		Layer		Treatment		Species × treatment		Layer × treatment		Species × layer		Treatment × species × layer	
	*F*-value	*p*-value	*F*-value	*p*-value	*F*-value	*p*-value	*F*-value	*p*-value	*F*-value	*p*-value	*F*-value	*p*-value	*F*-value	*p*-value
Root length	341.246	<0.01	427.818	<0.01	23.006	<0.01	13.105	<0.01	7.083	<0.01	26.574	<0.01	1.980	<0.01
Root surface	340.518	<0.01	405.624	<0.01	13.812	<0.01	8.601	<0.01	8.056	<0.01	20.433	<0.01	1.690	0.076
Specific root length	850.440	<0.01	260.549	<0.01	22.181	<0.01	9.959	<0.01	5.452	<0.01	71.718	<0.01	2.713	<0.01
Root dry weight	224.693	<0.01	1038.144	<0.01	5.617	<0.01	5.495	<0.01	3.941	<0.01	60.093	<0.01	0.803	0.647

## Results

### Seedling Growth

**Figure 2:**
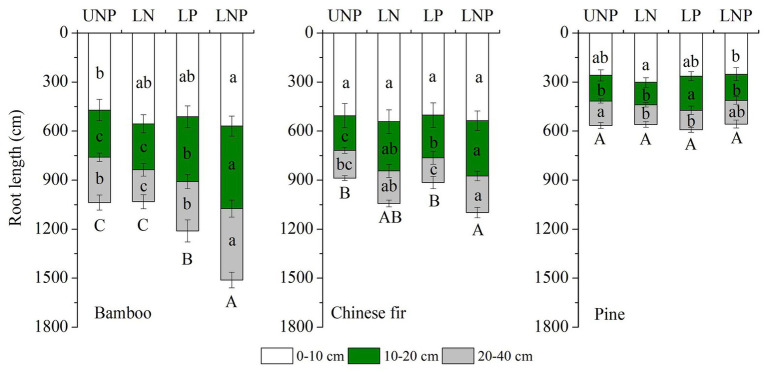
Effects of different nutrient supply treatments on the root lengths of the three tree species in each soil layer. Different lowercase letters denote significant differences within the same soil layer for each species among treatments (*p* < 0.05). Different capital letters denote significant differences in the total root length for each species among treatments (*p* < 0.05).

**Figure 3:**
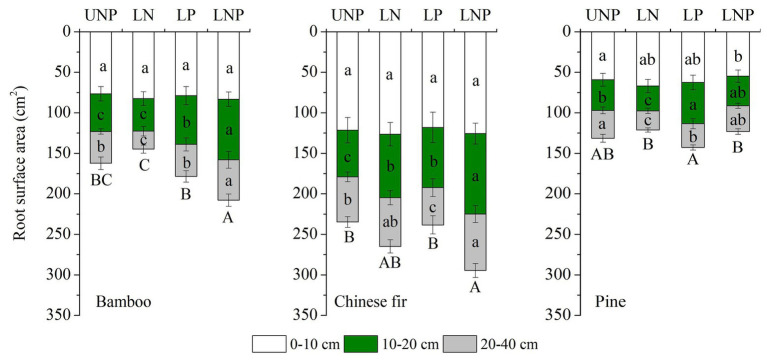
Effects of different nutrient supply treatments on the root surface areas of the three tree species in each layer. Different lowercase letters denote significant differences within the same category (soil layer) for each species among treatments (*p* < 0.05). Different capital letters denote significant differences in the total root surface area for each species among treatments (*p* < 0.05).

**Figure 4:**
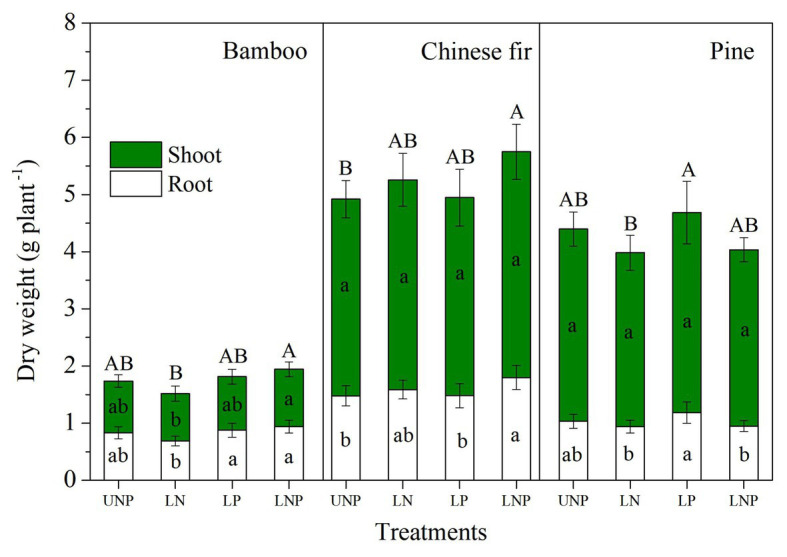
Shoot and root biomass of the three tree species in relation to different nutrient supply treatments. Different lowercase letters denote significant differences in shoot or root biomass within each species among treatments (*p* < 0.05), and different capital letters denote significant differences in total plant biomass within each species among treatments (*p* < 0.05).

The investigated root parameters were significantly affected by species, nutrient treatments, soil layers, and their interactions (Tables 1 and 2). Within each nutrient treatment type, the total root length showed a decreasing order as bamboo > Chinese fir > pine (Figure 2; Table 2), and the total root surface area followed an order of Chinese fir > bamboo > pine (Figure 3; Table 2), whereas the total plant biomass had an order of Chinese fir > pine > bamboo (Figure 4; Table 2). Differences among tree species are significant at the 0.05 level.

**Table 2:** Effects of species, nutrient treatments, and their interactions on whole plant and root parameters.

Parameter	Species		Treatment		Species × Treatment	
	*F*-value	*p*-value	*F*-value	*p*-value	*F*-value	*p*-value
Shoot biomass	350.648	<0.01	0.638	0.594	1.864	0.109
Root biomass (0–40cm soil layer)	126.729	<0.01	3.067	0.038	3.236	0.01
Total biomass	270.065	<0.01	1.274	0.295	2.337	0.048
Root-top ratio	1714.125	<0.01	4.745	<0.01	3.283	<0.01
NSC concentration in root	21.450	<0.01	3.951	0.014	4.620	<0.01
Total N pool size/plant	427.627	<0.01	1.217	0.315	4.261	<0.01
Total P pool size/plant	365.290	<0.01	3.384	0.026	2.549	0.033
Total root length (0–40cm soil layer)	164.542	<0.01	11.147	<0.01	6.335	<0.01
Total root surface area (0–40cm soil layer)	154.306	<0.01	6.253	<0.01	3.954	<0.01
Specific root length (0–40cm soil layer)	587.550	<0.01	7.586	<0.01	3.952	<0.01
Root length (10–20cm soil layer)	256.335	<0.01	32.866	<0.01	15.704	<0.01
Root surface area (10–20cm soil layer)	205.938	<0.01	29.361	<0.01	10.803	<0.01
Specific root length (10–20cm soil layer)	298.443	<0.01	4.779	<0.01	2.698	0.026

Compared with uniform fertilization (UNP), localized fertilization (LP and LNP) tended to increase the total root length (Figure 2) and total root surface area (Figure 3) of bamboo, while the treatments LN and LNP tended to increase the above two parameters of Chinese fir, whereas all of the three localized fertilization treatments did not affect the above two parameters of pine (Figures 2, 3). Treatment LNP increased the total biomass and root biomass of Chinese fir, while other localized fertilization treatments (LP and LN) showed no significant effect on the biomass of the three tree species (Figure 4).

### Root Morphology and Root Distribution

Root length and root surface area in the 10–20cm soil layer were significantly affected by species, nutrient treatments, and their interactions (Tables 1 and 2). Within the four nutrient treatments, root length and root surface area of bamboo in the 10–20cm soil layer showed a decreasing order of LNP > LP > UNP > LN (Figures 2, 3). Root length and root surface area of Chinese fir in the 10–20cm soil layer showed a decreasing order of LNP > LN > LP > UNP (Figures 2, 3). Root length and root surface area of pine in the 10–20cm soil layer showed a decreasing order of LP > LNP > UNP > LN (Figures 2, 3). It should be mentioned that treatment LP increased pine’ s root length and root surface area in the nutrient-enriched layer and decreased root length in the 20–40cm layer significantly, resulting in a non-significant change in total root length (Figures 2, 3).

**Figure 5:**
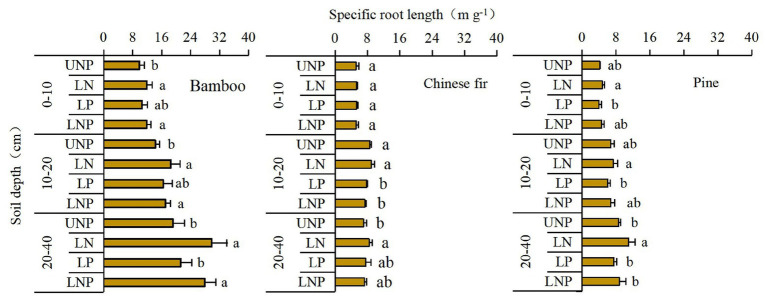
Effects of different nutrient supply treatments on the specific root lengths (SRLs) of the three tree species in each layer. Different lowercase letters denote significant differences among treatments within the same soil layer (*p* < 0.05).

Both total SRL (Table 1) and SRL in the 10–20cm soil layer were significantly affected by species, nutrient treatments, and their interactions. The SRL’s response to localized nutrient supply was also different among species. Bamboo SRL across the three soil layers was higher under LN and LNP than under UNP (Figure 5). For Chinese fir, compared with UNP treatment, the LNP treatment significantly reduced SRL in the 10–20cm layer only (Figure 5). Unlike in bamboo and Chinese fir, no significant difference between localized nutrient treatment and UNP was observed for the pine SRL in the 10–20cm layer.

### Root Response Ratio

**Figure 6:**
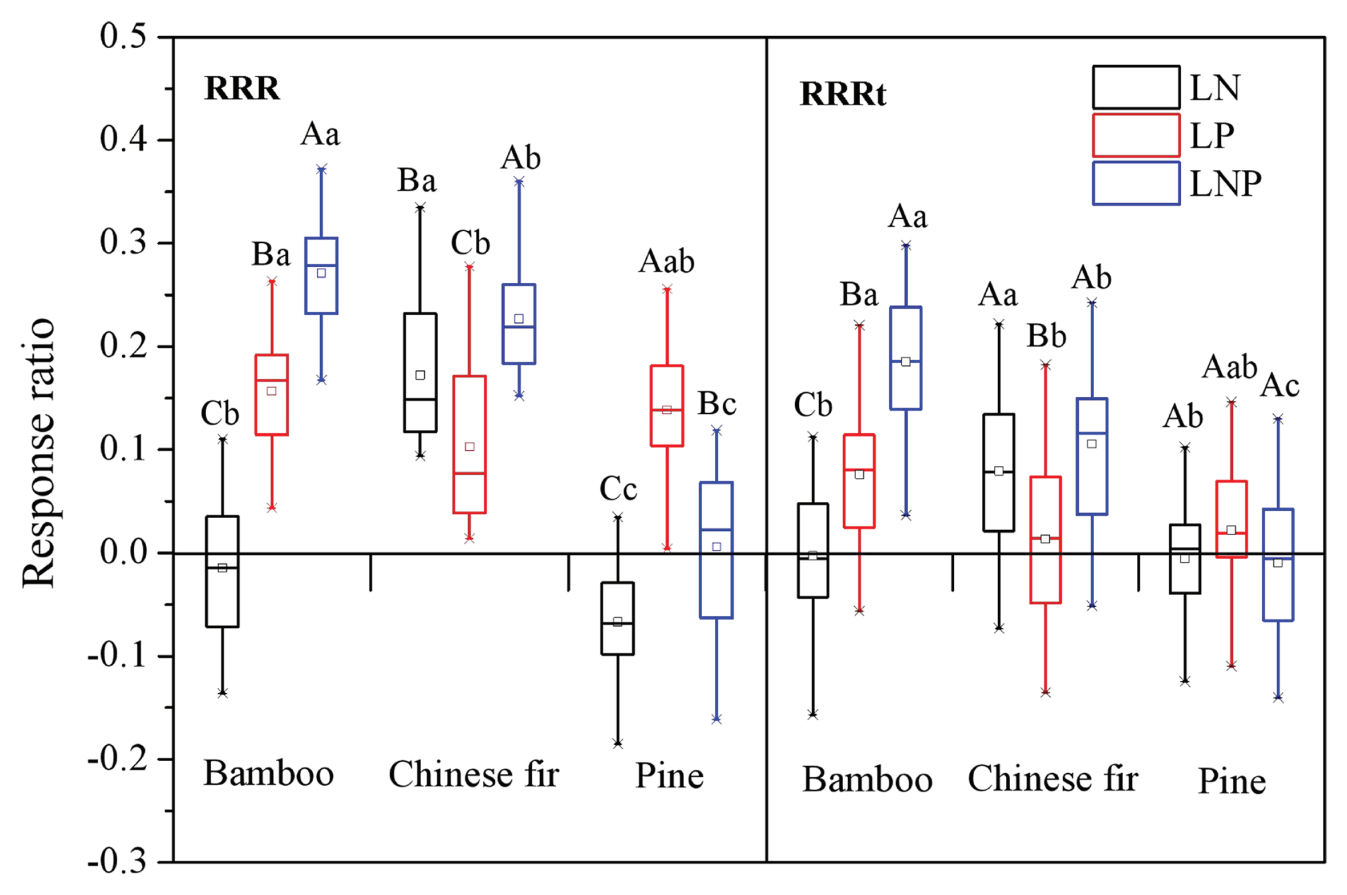
Response ratios (RRR and RRRt) under localized nutrient supply treatments (LN, LP, and LNP) in comparison to the uniform nutrient supply (UNP). Different lowercase letters denote significant differences among species (*p* < 0.05). Different capital letters denote significant differences among treatments (*p* < 0.05). RRR, root response ratio; RRRt, response ratio of total root length.

For each tree species, the RRR showed apparent nutrient-specific characteristic (Figure 6). The RRR of bamboo showed a decreasing order of LNP > LP > LN (*p* < 0.05), and that of Chinese fir followed an order of LNP > LN > LP (*p* < 0.05), whereas the RRR of pine followed the order LP > LNP > LN (*p* < 0.05; Figure 6). For every nutrient treatment, the RRR showed species-specific characteristics. Compared with bamboo and pine, the RRR of Chinese fir under LN treatment was relatively higher, while that under LP treatment was relatively lower. The RRR of the three species under the LNP treatment showed a decreasing order of bamboo > Chinese fir > pine (*p* < 0.05). The positive effect on bamboo and Chinese fir was enhanced when both elements were localized at the same time. For pine, however, the effect was offset with the RRR under LNP treatment being significantly lower than that under LP treatment but significantly higher than that under LN treatment (Figure 6).

A similar trend was observed in the RRRt results for all three species. The RRRt also showed species-specific and nutrient-specific characteristics. However, compared with the RRR, the absolute value of the RRRt under the same treatment was lower than that of the RRR, especially for pine (Figure 6).

### Foraging Precision and Foraging Scale

**Figure 7:**
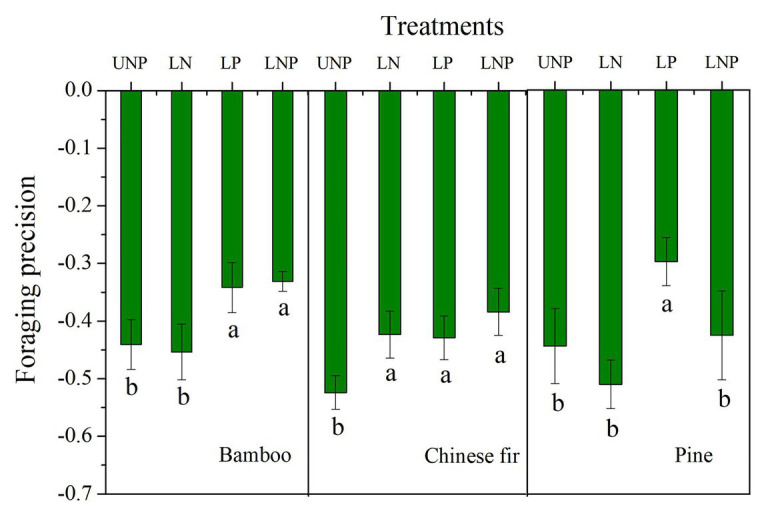
Effects of different nutrient supply treatments on the foraging precision of the three tree species. Different lowercase letters denote significant differences among treatments within the same soil layer (*p* < 0.05).

The FP of bamboo increased significantly under LP and LNP treatments, and that of Chinese fir increased significantly under all of the three localized fertilization treatments, while that of pine increased only under LP (Figure 7). The FS of bamboo increased significantly under LP and LNP treatments, and that of Chinese fir increased significantly under LNP (Figure 2).

### NSCs Concentrations and Nutrients Accumulation

**Figure 8:**
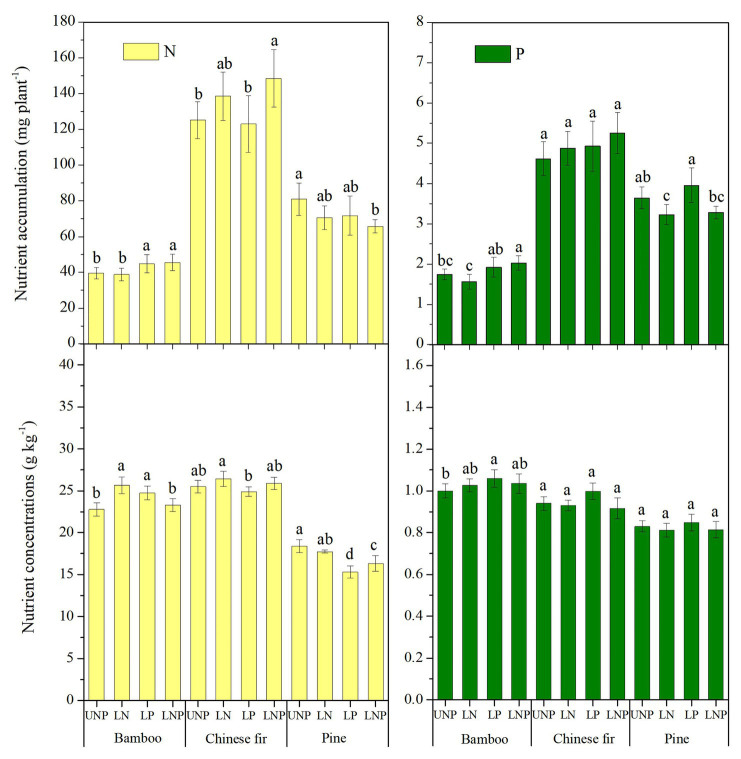
Total nutrients content and average nutrient concentrations of the three tree species under the four nutrient supply treatments. Different lowercase letters denote significant differences among treatments (*p* < 0.05).

Plant nutrient accumulation (total nutrient content) and NSC concentrations in roots were significantly affected by species, nutrient treatments, and their interactions (Table 2). Compared with UNP, LN significantly reduced the accumulation of N and P in pine, but it did not affect the accumulation of N and P in bamboo and Chinese fir (Figure 8). LP increased the N accumulation in bamboo significantly. LNP increased the N and P accumulation in bamboo and the N accumulation in Chinese fir but reduced the N accumulation in pine significantly (Figure 8). Consistent with the plant biomass (Figure 4), N and P accumulation also showed a decreasing order of Chinese fir > pine > bamboo (*p* < 0.05; Figure 8; Table 2).

Compared with UNP, LN significantly increased the N concentration of bamboo, while LP significantly increased the N concentration of bamboo and decreased the N concentration of pine, and LNP significantly decreased the N concentration of pine. In addition, LP increased the P concentration of pine and Chinese fir, although the effect was not significant (Figure 8).

**Figure 9:**
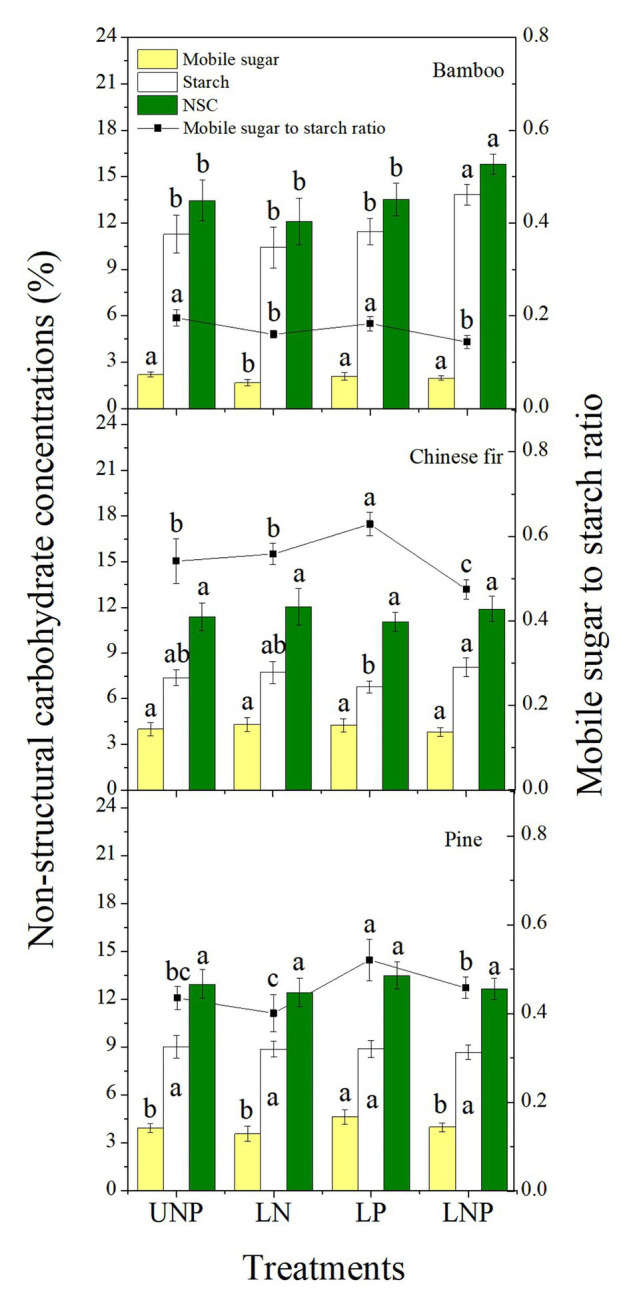
Concentrations of non-structural carbohydrates (NSCs) and mobile sugar to starch ratio in roots of the three tree species grown under the four nutrient supply treatments. Different lowercase letters denote significant differences within the same category among treatments (*p* < 0.05). NSC, non-structural carbohydrate.

Compared with UNP, LN significantly decreased the concentrations of mobile sugars and reduced the sugar-starch ratio in bamboo roots. The LP treatment significantly increased the mobile sugar-starch ratio in Chinese fir roots and significantly increased the mobile sugar concentration and the mobile sugar-starch ratio in pine roots. The LNP treatment significantly increased the starch concentration and decreased the sugar-starch ratio in bamboo and Chinese fir roots and significantly increased the NSC concentration in bamboo root (Figure 9). The three species had similar root NSC concentrations, but their sugar-starch ratios had a decreasing order of Chinese fir > pine > bamboo (*p* < 0.05; Figure 9; Table 2).

### Correlations Between Root Morphology and Resources

The root morphology (root length and root surface area) of both bamboo and Chinese fir was positively correlated with the concentrations of starch and NSC, total N, and total P but negatively correlated with mobile sugar-starch ratio. Root growth (root length and root surface area) of pine was positively correlated with mobile sugar, concentration, total N, total P, and mobile sugar-starch ratio (Table 3).

**Table 3:** Analysis of Pearson correlations for the localized nutrient treatments.

Species	Parameter	Mobile sugar	Starch	NSC	Mobile sugar/starch	Total N	Total P
Bamboo	Root length	0.190	0.671^**^	0.651^**^	−0.421	0.757^**^	0.806^**^
	Root surface area	0.314	0.593^**^	0.598^**^	−0.216	0.866^**^	0.908^**^
	RRR	0.413	0.654^**^	0.658^**^	−0.241	0.701^**^	0.815^**^
	RRRt	0.398	0.648^*^	0.650^*^	−0.256	0.755^**^	0.848^**^
Chinese fir	Root length	0.283	0.501^*^	0.518^*^	−0.135	0.888^**^	0.700^**^
	Root surface area	0.137	0.410	0.380	−0.192	0.938^**^	0.783^**^
	RRR	0.146	0.657*	0.617*	−0.392	0.895^**^	0.630^*^
	RRRt	0.288	0.560^*^	0.601^*^	−0.199	0.909^**^	0.689^**^
Pine	Root length	0.111	0.200	0.191	0.025	0.295	0.317
	Root surface area	0.360	0.225	0.358	0.283	0.554^*^	0.743^**^
	RRR	0.458	−0.110	0.251	0.530	0.047	0.551^*^
	RRRt	0.136	0.048	0.118	0.127	0.273	0.262

RRR, root response ratio; RRRt, response ratio of total root length; NSC, non-structural carbohydrate.

* Denotes a significant correlation between two parameters (*p* < 0.05).

** Denotes a significant correlation between two parameters (*p* < 0.01).

Within the three localized nutrient treatments, the RRR and RRRt of both bamboo and Chinese fir were positively correlated with the starch and NSC concentrations, total N and total P. For pine, significant positive correlation was found only between RRR and total P (Table 3).

## Discussion

### Species-Specific and Nutrient-Specific Foraging Strategies

The three examined tree species showed different responses in their foraging strategies with regard to the localized nutrient supply. Bamboo and pine responded to LP by increasing the root length in P-rich patch but showed a negative response to N-rich patch, indicating that the sensitivity of pine and bamboo roots to P was significantly stronger than that to N (Figures 2, 3). The opposite result was found in the roots of Chinese fir. The ability of roots to proliferate in nutrient-rich patches depend on plant demand for, sensitivity to, and tolerance to, specific elements (Hodge et al., 1998; Fransen and de Kroon, 2001; Hodge, 2004; Li et al., 2014). Chinese fir is more sensitive to N-rich patch than bamboo and pine, which may result from its higher total N and N content per unit dry weight (Figure 8). Studies have shown that a high concentration of N inhibits lateral root elongation and proliferation (Nakaji et al., 2001; Joshi et al., 2016). Increase in root respiration (Hoch and Körner, 2003) and the changes in signaling pathway of abscisic acid and auxin (Bishopp et al., 2011; Lavenus et al., 2013) owing to high N levels may ultimately hinder root growth. In addition, plants may show low root plasticity as a result of their excellent capturing ability for specific elements, such as leguminous and ECM tree species (Harrison and Van Buuren, 1995; Lopez-Pedrosa et al., 2006; Hijikata et al., 2010; Li et al., 2014).

The responses of Chinese fir and bamboo roots to localized nutrient supply were more intense than those of pine (Figure 6). According to previous studies, significant differences in root responses to nutrients between tree species depended on the growth rate (Fitter, 1994; Garnier et al., 2004), SRL (Eissenstat et al., 2015), and mycorrhizal type (Cheng et al., 2016). Roots with low SRL tend to be less flexible during foraging because the cost of root elongation in nutrient patches is high (Eissenstat, 1991; Mou et al., 1997; Li et al., 2014). The characteristics of these species do not fully explain the results of this study. Difference root architecture of the three species may also be responsible for the difference of root plasticity. Pine is a typical taproot tree species with a distinct primary root and sparse lateral roots. This branching mode is costly, but its lower competition strengthens the plants’ viability in nutrient-deficient soil. The low root plasticity of pine in nutrient heterogeneity soil may be due to the high forming cost of its root (Fitter et al., 1991; Bouma et al., 2001; Sorgonà et al., 2005; Lynch, 2013). In contrast, both bamboo and Chinese fir are typical fibrous root species with well-developed lateral roots and no apparent taproot. This low-cost branching mode results in the ability to occupy soil space quickly and is more suitable for nutrient-rich soils (Fitter et al., 1991; Bouma et al., 2001; Tsakaldimi et al., 2009; Fry et al., 2018). Therefore, the different types of root branching may be an important factor in determining the plasticity of bamboo and Chinese fir roots compared with pine roots. In addition, the role of mycorrhizas should not be ignored because mycorrhizas provide a larger absorption area for most tree species (over 90%; Brundrett, 2009; Bonfante and Genre, 2010). The absorption abilities of mycorrhizas and the morphology of roots are often complementary. Thicker roots tend to rely more on the absorption of mycorrhizas, lessening their morphological plasticity (Hoeksema, 2010; Liu et al., 2015; Cheng et al., 2016). Compared with AM tree species, ECM tree species rely more on absorption by mycorrhizas. Trees tend to balance the distribution of carbon between mycorrhizas and roots. The response of ECM tree species to nutrient hotspots is often reflected in the mycorrhizas and these species have morphological root plasticity levels lower than those of AM tree species (Read and Perez-Moreno, 2003; Liu et al., 2015; Cheng et al., 2016). Fast-growing trees tend to exhibit higher root plasticity to adapt to nutrient heterogeneity than slow-growing trees (Van de Vijver et al., 1993; Fransen et al., 1999). The differences of foraging strategies of the three co-existing tree species in this study may be used to explore the methods of tree species allocation and fertilization with higher soil space and resource utilization efficiency.

The FP of all three tree species under the four treatments was negative, mainly because more soil space belonged to nutrient-poor layer. The root systems of the three species were unevenly distributed in the vertical direction of soil, i.e., more roots of the three species distributed in the topsoil. The FP is majorly positive when the nutrient patches are distributed horizontally (instead of vertically) and the different patches sizes are equal (Wijesinghe et al., 2001; Chen et al., 2018). Increasing of FP in nutrient heterogeneity soils is common in previous studies, including clonal plants, woody, and herbaceous plants (Wijesinghe and Hutchings, 1997; Stevens and Jones, 2006). However, the root distribution and FP of some tree species are not affected by nutrient heterogeneity soils, and even the root length was lower in nutrient-rich patch than in nutrient-poor patch (Wijesinghe et al., 2001; Wu et al., 2011). Leading factors of this species-specific foraging strategies having been suggested include tree size, growth rate, and the balance between FP and FS (Fransen et al., 1999; Wijesinghe et al., 2001; Chen et al., 2018). The response of the total root length to the localized nutrient supply was generally not as strong as that of root length in the nutrient-enriched layer, i.e., the foraging precision increased for the three tree species in heterogeneous soil (Figures 6, 7). For instance, LP treatment increased root length and root surface area of pine in the nutrient-enriched layer, but decreased root length and surface area in the 20–40cm layer, resulting in no significant change in total root length. This made it possible to increase the efficiency of carbon use by allocating a greater proportion of the carbon to the roots in the nutrient patches (Hodge, 2009; Chen et al., 2016). This strategy of root distribution, similar to the compensatory effect, has been found in previous studies (Drew, 1975; Granato and Raper, 1989; Hodge, 2009). However, unlike previous studies on compensatory effects, the reduction of root length occurred in the nutrient-deficient layer below the nutrient-enriched layer and not in the nutrient-deficient layer above the nutrient-enriched layer (Li et al., 2014). The transport of photosynthates from aboveground parts to roots depends on the concentration difference between sinks and sources (Koch and Avigne, 1990; Oparka and Turgeon, 1999; Chen et al., 2017a). For the LP treatment, a large amount of carbon was consumed by the root expansion in the nutrient-enriched layer, which may reduce the concentration difference between the nutrient-enriched layer and the distal roots and thus inhibited carbon input in the roots in the 20–40cm layer.

In this study, the negative effects of the N concentration on pine root growth were alleviated by the localization of P, compared with localization of a single element (LP and LN), LNP showed a more significant effect on the root growth of bamboo and Chinese fir in the nutrient-enriched layer, indicating that N and P often exhibit synergistic effects on root formation (Li et al., 2012; Huysen et al., 2016). This phenomenon of the interaction between elements may be related to the change in soil physical and chemical properties. It has been suggested that the absorption of ammonium N by roots will reduce the soil pH and improve the availability of P in the soil (Jing et al., 2010; Li et al., 2014). Some studies have shown that P addition can activate N by changing the soil physical and chemical properties, microbial and soil enzyme activities (Li et al., 2012; Huysen et al., 2016).

### Root Formation Strategy

Bamboo root quickly occupies soil space of nutrient patches at lower cost relying on the formation strategy, which increases its length and SRL in nutrient patches. A similar result was reported by Nakamura et al. (2008) that *Lolium perenne* roots increased its SRL and elongated both laterally and vertically in nutrient heterogeneous soils. Bamboo also increased the root length and SRL in the 20–40cm layer. This forced more fine roots to be generated in nutrient-deficient areas, thus allowing for the occupation of a wider soil space at a lower structural cost. These responses could improve the probability of roots’ encountering nutrient-rich patches and the efficiency of nutrient acquisition from nutrient-poor regions outside the patches. In contrast, LNP induced Chinese fir root thickening and decreased SRL in the nutrient-enriched layer. This strategy of increasing the cost of root construction in nutrient-enriched areas to avoid more branches of roots and thus reduce competition within the root system is considered beneficial to long-term root development (Fransen and de Kroon, 2001; Garnier et al., 2004; Saengwilai et al., 2014; Li et al., 2017). To sum up, the root formation strategy of moso bamboo in nutrient patches is to increase SRL, reduce root construction cost and respiration consumption and to improve carbon use efficiency, while that of Chinese fir reduces SRL and internal competition and increases absorption area. This opposite result may be related to the ability of plants to balance root metabolism and root absorption (Miller et al., 2003; Walk et al., 2006). The fibrous root species with well-developed lateral roots are more dependent on this regulatory balancing ability. The inflexibility of SRL in nutrient-rich layer indicates pine’s weak ability to improve its nutrient absorption efficiency by changing root formation strategy. In summary, it suggests that root foraging strategies in nutrient patches include not only rapid occupation of nutrient patches through rapid proliferation, but also changes in root formation strategies, which are species-specific.

### Contributions of Photosynthates to Root Plasticity

The concentration of NSC in plant tissues is closely related to root proliferation (Horwath et al., 1994; Bell and Ojeda, 1999; Gaudinski et al., 2009). Changes in NSC level reflect not only the plants physiological activity responses to environmental changes but also the carbon balance between structural growth and respiratory consumption (Ericsson et al., 1996; Li et al., 2002; Rodríguez-Calcerrada et al., 2016). The concentration of NSC in the roots of bamboo was significantly increased by the LNP treatment (Figure 9) and correlated positively with root growth and RRR, confirming previous views (Table 3). It suggests that the root morphology plasticity of bamboo profits from the rapid change in its NSC concentration. In addition, responses of NSC concentration to nutrient heterogeneity varied from species to species, which may have led to the significant differences in root plasticity and root formation strategies between the three species in response to soil nutrient heterogeneity (Figure 9). In bamboo and pine, the reduction of NSCs induced by LN treatment may have been caused by the effect of high N on the hormones (ABA and IAA) in the root tissue, which affected the activity of ATPase and thus inhibited assimilate absorption by the sink cells (Clifford et al., 1986; Peng et al., 2003). The LP treatment significantly increased the concentration of root soluble sugars and the ratio of mobile sugars to starch in the roots of pine and Chinese fir (Figure 9). One possible explanation would be that an increase in P content in root tissue promotes the accumulation of NSCs and the activity of *α*-amylase, thereby promoting the degradation of starch in storage cells, which may eventually lead to a significant increase in the ratio of sugar to starch in the pine and Chinese fir roots treated with LP (Gallagher et al., 1997; Li et al., 1998).

### Contribution of Root Plasticity to Nutrient Accumulation

The contribution of root plasticity to nutrient accumulation was also different for the three examined tree species. The contribution of the morphological plasticity of bamboo and Chinese fir roots to nutrient accumulation was obviously greater than that of pine. Tree species with better root plasticity had an advantage expressed in increased aboveground growth and nutrient accumulation, which support those reviewed by Wang et al. (2018). The nutrient accumulation of the Chinese fir and bamboo as well as the biomass of Chinese fir all benefited from nutrient localization in this experiment (Figures 4, 7). However, it has been reported that localized nutrient supply does not affect the plant growth and nutrient accumulation significantly when the total nutrient content of soil is constant (He et al., 2012; Li et al., 2014). The difference between our study and previous studies may be due to the different durations of the experiment. The effect of a localized nutrient supply on nutrient and dry matter accumulation disappears with the prolongation of the trial period and the depletion of nutrients (Jing et al., 2010; Li et al., 2012). In addition, the rhizome system of moso bamboo may develop over time and may show different foraging strategy from that of its seedlings. Therefore, future research should determine the responses of tree species to soil with heterogeneous nutrients over different time scales.

In this experiment, nutrient localization did not have a positive effect on nutrient accumulation in pine. Zhang et al. (2013) obtained the opposite result: the root growth and nutrient accumulation of masson pine in a heterogeneous low-P condition with high P in the top layer were significantly greater than those in low P environment during local P treatment. One possible explanation is that the total amount of soil nutrients in the four treatments in this study is the same. The increase of FS in competition for nutrients seems to be more effective than the increase of FP. The LN and LP treatments increased the FP of Chinese fir and did not increase the FS, which resulted in no significant change in nutrient accumulation of Chinese fir. Similarly, the pine only increased the FP instead of increasing the FS and nutrient accumulation like bamboo (Figure 7). It suggests that plants cannot increase their biomass and nutrient accumulation by merely increasing FP in nutrient patches without FS increase. Chen et al. (2018) found a similar conclusion that the increase of FS was more favorable for nutrient absorption than the increase of FP. On the contrary, Li et al. (2012) suggested that the contribution of root plasticity to nutrient uptake depends on the length proportion of roots in nutrient patches, i.e., FP. In fact the increase in FS and FP will make plants more competitive, even if the accumulation of biomass and nutrients does not change in a short time (Rajaniemi, 2007). However, if FS and FP are not increased in nutrient patches, nutrient localization would not be conducive to nutrient uptake due to the decrease of foraging space, for example, the LNP treatment reduced total N and P of pine in this study (Figure 7).

## Conclusion

In line with our hypotheses, we found that the root plasticity of the fibrous root species moso bamboo and Chinese fir was greater than that of the taproot species masson pine. Chinese fir tended to forage more N by promoting root proliferation in the N-rich patch, while root proliferation of bamboo and pine did not change. For P, bamboo absorbed more nutrients by promoting root proliferation in the P-rich patch. Chinese fir foraged more N by increasing root length and decreasing SRL in the NP-rich patch; bamboo foraged more N and P by increasing root length and SRL in the NP-rich patch. Moso bamboo and Chinese fir increased their FS and foraging precision, while masson pine increased their foraging precision only. Therefore, the root foraging strategies of the three species in nutrient heterogeneous soils are species-specific and nutrient-specific. These results reflect the differences in the biological properties among co-existing species. Our findings also provide basic knowledge for improving fertilization management practices and increasing the foraging precision as well as the soil nutrient utilization efficiency in mixed forests. The NSC concentration in the roots of bamboo and Chinese fir was highly related to the morphological changes (RRR) caused by nutrient localization, while it was not observed in pine. Photosynthates contributed more to the root morphological plasticity of bamboo and Chinese fir than that of pine. This result provides insights for further exploring the physiological mechanisms supporting root plasticity.

## Data Availability Statement

The raw data supporting the conclusions of this article will be made available by the authors, without undue reservation.

## Author Contributions

ZY and BZ designed the experiments. ZY, BZ, M-HL, JS, XG, and YC performed the analysis. ZY, JS, BZ, IB, and M-HL drafted the manuscript. All authors critically revised and approved the final version of this manuscript.

### Conflict of Interest

The authors declare that the research was conducted in the absence of any commercial or financial relationships that could be construed as a potential conflict of interest.
